# Creative Arts Interventions as a Way to Combat Ageism and Increase Student Interest in Gerontology

**DOI:** 10.1093/geroni/igab046.1549

**Published:** 2021-12-17

**Authors:** Emily Ihara, Catherine Tompkins, Megumi Inoue, Kendall Barrett

**Affiliations:** George Mason University, Fairfax, Virginia, United States

## Abstract

Despite the continuous, growing need for health professionals who are trained to work with older adults living with Alzheimer’s disease and related disorders (ADRD), research shows that recruitment and retention of gerontological health care professionals remains low. Ageism plays an important role in this resistance and continues to have societal impact, even proliferating in disturbing ways during the COVID-19 pandemic via stereotypes, discrimination, and framing in the media. Gerontologists in various health professional educational settings continue to address the need to infuse aging content in creative ways and increase the competency of all health professionals to combat ageism and understand the importance of specialized care for this population. Our gerontological research team has engaged students in various ways to increase interest in aging issues and ADRD. Current research projects involve the implementation of non-pharmacological, creative arts interventions, including Mason’s Music & Memory Initiative (M3I) and TimeSlips, both which have the potential to appeal to intergenerational partnerships and provide students with tools to communicate better with those living with ADRD. We examined attitudes about aging among undergraduate and graduate students (N=78) who have completed our training modules and/or participated in these two projects. The asynchronous trainings provide content on ADRD and the implementation of non-pharmacological, creative arts interventions. We examined students’ attitudes about aging and ADRD and analyzed their open-ended responses regarding their experiences with someone living with ADRD. Various levels of education, relationships with older adults, and life experience influenced their responses regarding their attitudes about aging.

